# The Effectiveness of Counseling in Reducing Anxiety Among Nulliparous Pregnant Women 

**Published:** 2016-12

**Authors:** Parisa Parsa, Nafiseh Saeedzadeh, Seyedeh Zahra Masoumi, Ghodratallah Roshanaei

**Affiliations:** 1 Chronic Diseases (Home Care) Research Center, Department of Mother and Child Health, Hamadan University of Medical Sciences, Hamadan, Iran; 2 Department of Midwifery, Student Research Center, Hamadan University of Medical Sciences, Hamadan, Iran; 3 Department of Midwifery, Mother and Child Care Research Center, Hamadan University of Medical Sciences, Hamadan, Iran; 4 Department of Statistics, Faculty of Public Health, Hamadan University of Medical Sciences, Hamadan, Iran

**Keywords:** Fetus, Anxiety, Pregnancy, Consultation, Mother

## Abstract

**Objective:** To determine the effectiveness of counseling in reducing anxiety of nulliparous pregnant women.

**Materials and methods:** In this quasi-experimental study, 110 nulliparous pregnant women were selected out of all pregnant women referring to Fatemieh Hospital in Hamadan, Iran. Then, the subjects were divided into two groups in experimental and control (55 women in each). The data were collected through a questionnaire covering demographic and obstetric characteristics and Spielberger’s State-Trait Anxiety Inventory. The experimental group participated in four weekly sessions of group counseling about mother-infant attachment behaviors. Whereas, the control group only receive routine cares. Two groups were compared in terms of anxiety before and after the study.

**Results:** Before the intervention, no significant difference in anxiety level was observed between the two groups; however, state and trait anxiety levels of pregnant women in the experimental group significantly decreased after the intervention (p < 0.001). There was also significant difference in the mean score of state and trait anxiety levels between the two groups after the intervention (p < 0.001).

**Conclusion:** The results showed the effectiveness of prenatal counseling in reducing state and trait anxiety levels of pregnant women.

## Introduction

Pregnancy and motherhood are the most gratifying and evolutionary stages of women’s lives. These stages are associated with physiological and psychological changes requiring special attention (1). For many women, pregnancy creates psychological problems such as anxiety, depression and feeling of uncertainty in life (2, 3). How to grow a fetus in the mothers’ womb can predict the state of one's health later in life. As a stressful stimulus, mothers’ anxiety during pregnancy can cause future mental and psychological problems in newborns (4, 5). Such mental and psychological problems have been observedin both newborns and adults (6). Mother’s anxiety in the prenatal period can cause 10-15% of behavioral and emotional disorders in babies’ lives. Among those disorders, Attention Deficit and Hyperactivity Disorder (ADHD) and cognitive problems can be mentioned (3, 6). Psychobiological factors of mothers’ anxiety during pregnancy can lead to delayed fetal maturation and mood disorder, learning disability and memory loss in children aged 6-8 years (7). This is not yet clear how mothers’ anxiety during pregnancy can cause psychological disorders in their children; but, one hypothetical cause is prenatal exposure to excessive glucocorticoids levels. Hypothalamic-Pituitary-Adrenal (HPA) axis dysfunction in anxious mothers and their fetuses has been considered responsible for future behavioral disorders in children (6). Prenatal exposure to cortisol can have negative effects on children’s cognitive abilities in the future (8, 9). Normally, pregnant women become more aware of their fetuses at the end of the second trimester; thus, bonding with their unborn babies become more valuable to mothers and this relationship enhances their mood and feelings (10). On the other hand, it has been shown that a reduction in the amount of cortisol in pregnant women’s bloodstream can prevent excessive levels of glucocorticoids in fetal HPA axis (11). Highly anxious pregnant women have also problems in bonding with their babies (12). The primary relationship between mother and fetus during pregnancy is called ‘maternal-fetal attachment’ (12). Women’s anxiety increases during pregnancy, especially in the final days. Therefore, childbirth education helps mothers experience a healthier pregnancy with fewer complications and leads to reduced levels of pain, anxiety and depression in pregnancy (5). Among the interventions used to enhance maternal-fetal attachment is attachment behavior education and consultation that include talking to the fetus, touching the abdomen, paying attention to fetal movements, etc. It seems that such behaviors promote maternal-fetal attachment and increase mothers’ attention to their fetuses (13). Maternal-fetal attachment is a mother’s abstract idea about her fetus that potentially exists before the childbirth. This abstract idea is associated with emotional and cognitive processes in relation to the perception of another human being’s existence. According to John Bowlby, maternal-infant attachment develops long before birth. He believed that this attachment relationship is essential for survival (10). On the other hands, the relationship between prenatal exposure to cortisol and children’s future cognitive abilities can probably be affected by the level of maternal-fetal attachment (8). Mental health care is one of the important aspects of pregnancy care programs (3). Lack of sufficient support from family members and health care workers may result in increased levels of anxiety in pregnant women (4). Strengthening prenatal attachment improves pregnancy health care and facilitates mothers’ compliance with the role of mother in the future (11, 13).

Some studies have indicated that maternal-fetal attachment behaviors education can lead to better interactions between mothers and their fetuses in prenatal periods and better relationships between mothers and their children after childbirth (14-18). However, Aheren and Ruland stated that there is no relationship between mothers’s behaviours such as touching the fetus from the abdomen and attachment to their babies after the childbirth (19). In fact, there are conflicting results regarding the effects of mother-fetal attachment behaviors. Accordingly, the present study was conducted to examine the effect of prenatal counseling about attachment behaviors on pregnant women’s anxiety.

## Materials and methods

The present quasi-experimental study was conducted on 110 eligible pregnant women selected out of all pregnant women referring to Fatemieh Hospital in the city of Hamadan, Iran in year 2015. 

**Figure 1:**
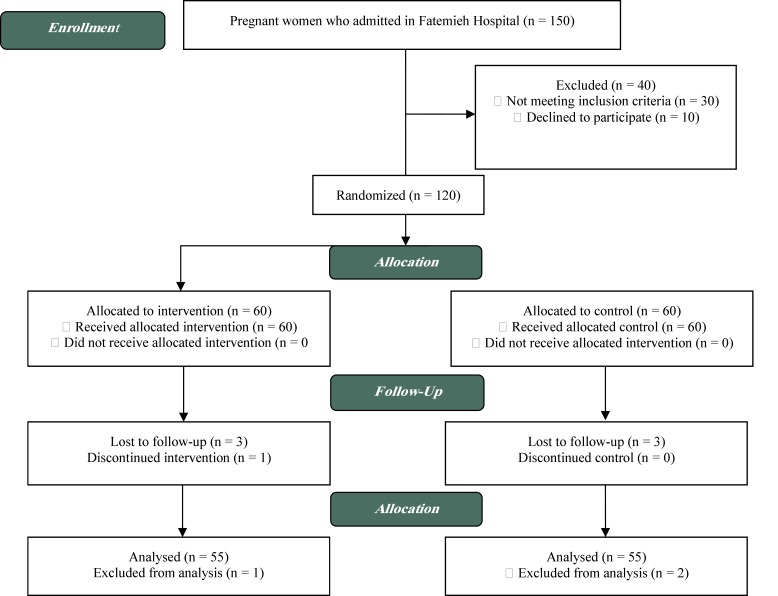
Flowchart of the study

The women were selected randomly to receive either consultation and routine cares or only routine care. They were selected based on a convenient sampling method and were randomly allocated into experimental and control groups. So that 120 pregnant women, 30-32 weeks pregnant women who had met the study’s criteria were assigned to either experimental or control group using permuted-block randomization design. The allocation sequence was determined by one of the members of the research team, not involved in the sample selection, using a four-block randomized design. The participants were randomly assigned to the intervention and control group with a ratio of 1:1. The codes related to each woman were specified in envelopes in order not to expose the assignment procedure. Thus, the subjects were in A and B group according to the specified sequence. The CONSORT 2010 flow diagram for the study is given in Figure 1.Therefore, if A was the experimental group and B was the control group, the participants would be divided by using randomized blocks (ABBA). The data analyzer was not aware of the interventions provided to the groups. Finaly 55 pregnant women were analyzed in the experimental group and 55 to the control group (Figure 1).

Every participant signed an informed consent form and had the right to stop participating at any stage of the study. A basic demonstration was presented to mothers for two groups. Eligible participate mothers, achieved a clear understanding of the relevant facts, risks and benefits, and available alternatives involved. The concept of informed consent originated with the recognition that participants have rights: to autonomy, human dignity and freedom. Mothers (whether in experimental or control) possess these rights and cannot be denied their rights due to mental health status or condition.

The inclusion criteria included being a first-time mother, gestational age of 30 to 32 weeks, lack of risky medical or obstetric problems, such as diabetes, preeclampsia, bleeding and Premature Rapture of Membranes (PROM), and having either a low or an average level of anxiety based on the STAI scores (i.e. STAI scores between 20 and 42). The exclusion criteria included any type of risky medical condition and unwillingness to participate. The needed data were collected through a questionnaire consisting of two parts: the first part included demographic characteristics of the participants, such as, age, education level, occupational status, wanted/unwanted pregnancy, duration of marriage, spouse’s education level and occupational status plus some prenatal information; the second part included Spielberger’s STAI that has been translated in 48 languages (20).

Reported Cronbach’s alphas for the STAI vary between 0.86 to 0.93 (21). Mahram standardized the STAI on 600 Iranian people and found reliability coefficients of 0.91, 0.90 and 0.94, respectively for state anxiety, trait anxiety and the total scale (22). The STAI consists of 20 questions evaluating how respondents feel ‘right now’ (state anxiety) and 20 questions measuring how they feel ‘generally’ (trait anxiety). The scale is scored on a 4-point Likert scale ranging from 1 (not at all) to 4 (very much so) for state anxiety items and from 1 (almost never) to 4 (almost always) for trait anxiety items; thus, the range of possible scores in each subscale is between 20 and 80 (15).

The study’s objectives and processes were explained to the participants before signing their informed consent forms. Then, they were asked to complete the study’s questionnaire before and after the intervention. The participants’ anxiety scores were calculated and pregnant women were matched in terms of participating in physiologic childbirth classes. The intervention consisted of four 45-minute counseling sessions. Participants in the experimental group were divided into two 25-member groups for participating in counseling sessions. The counseling sessions were designed based on the GATHER approach (23): G stands for ‘greet’ that is building a rapport with clients by greeting them and making them feel comfortable; A stands for ‘ask’ that is asking relevant questions about the clients’ concerns and identifying their needs; T stands for ‘tell’ that is helping clients by providing them with relevant information to help them reach appropriate decisions and make informed choices (in the present study, the participants were provided with information about the effectiveness of maternal-fetal attachment in reducing anxiety levels); H stands for ‘help’ that is helping the clients to reach a decision (in the present study, the participants learned how to communicate with their fetuses); E stands for ‘explain’ that is explaining about the method’s characteristics (the participants in the present study were explained about effective attachment techniques); finally, R stands for ‘return’ emphasizing the need for follow-ups (at the end of the intervention and two weeks later, the participants in both groups (i.e. 36 or 38 weeks pregnant women) were asked to complete the STAI again. Then, they were compared in terms of their STAI scores. In the first counseling session, pregnant women were educated on pregnancy-related physiological and hormonal changes and their effects on mind and body. They also learned strategies for coping with those changes; for example, they learned maternal-fetal attachment techniques (e.g. touching the abdomen and being familiar with the fetus) and understood their benefits. In the second counseling session, pregnant women were educated about nutrition during pregnancy and fetal developmental stages. Some other attachment techniques such as, counting and recording fetal movements and positively imagining the appearance of fetus, were also taught to the participants and practiced by them in the second session. In the third counseling session, pregnant women were educated on pregnancy warning signs and ways to deal with them. Moreover, the participants practiced attachment techniques, such as, paying attention to fetal movements, touching the abdomen, talking to the fetus, calling its name, looking at the abdomen, etc. in the third session. The last session included focusing on the fetus, touching the fetus from the abdomen and guessing its position, calming the fetus by touching the abdomen. In the final session, the participants also learned about stages of the birth process, breastfeeding and hugging newborns (13). 

The collected data were analyzed using the SPSS-16 software. Measures of central tendency and dispersion were used to extract descriptive information and paired and independent t-tests were used to compare groups.

## Results

The results showed no significant difference between the two groups in demographic factors of mothers’ and husbands’ average ages, education levels and occupational statuses (Table 1). Independent t-test was used to investigate the difference between the two groups in trait anxiety levels before and after the intervention. As indicated in Table 2, before the intervention, no significant difference was observed in trait anxiety between the two groups of experimental and control. However, a significant difference was found in trait anxiety level between the two groups after the intervention.

Paired t-test was conducted to investigate the difference in trait anxiety level in each group before and after the intervention. As indicated in Table 2, trait anxiety levels of pregnant women significantly changed as a result of intervention (p < 0.001). However, no significant difference was found in trait anxiety levels of pregnant women in the control group before and after the intervention.

**Table 1:** Comparison of demographic factors in two groups

Characteristics		Intervention n (%)	Control n (%)	p
**Women’s education level**	**Primary**	**2 (3.6)**	**8 (14.5)**	**0.190**
	**Secondary**	**24 (43.6)**	**18 (32.7)**	
	**Tertiary**	**29 (52.7)**	**29 (52.7)**	
**Spouses’ education level**	**Primary**	**3 (5.5)**	**10 (18.2)**	**0.24**
	**Secondary**	**23 (41.8)**	**22 (40.0)**	
	**Tertiary**	**29 (52.7)**	**23 (41.8)**	
**Women’s age (year)**	**18-22**	**9 (16.4)**	**3 (5.5)**	**0.30**
	**23-27**	**26 (47.3)**	**27 (49.1)**	
	**28-32**	**16 (29.1)**	**20 (36.4)**	
	**33-37**	**4 (7.3)**	**5 (9.1)**	
**Spouses’ age**	**23-27**	**17 (30.9)**	**13 (23.6)**	**0.60**
	**28-32**	**21 (38.2)**	**24 (43.6)**	
	**33-37**	**17 (30.9)**	**18 (32.7)**	
**Women’s job status **	**Housewife**	**48 (87.3)**	**48 (87.3)**	**0.99**
	**Employed**	**7 (12.7)**	**7 (12.7)**	
**Spouses’ job status**	**Employed**	**22 (40.0)**	**22 (40.0)**	**0.99**
	**Self-employed**	**60 (33.0)**	**60 (33.0)**	

As indicated in Table 3, before the intervention, no significant difference was observed in trait anxiety between the two groups of experimental and control. Paired t-test was conducted to investigate the difference in state anxiety level in each group before and after the intervention. As indicated in Table 3, state anxiety levels of pregnant women significantly changed as a result of intervention (p < 0.001). However, no significant difference was found in state anxiety levels of pregnant women in the control group before and after the intervention.

According to the results, the examined pregnant women used the following sources of information about pregnancy: physicians (42.7%), books, journals and the internet (41.8%), nurses and hospital personnel (38.2%), parents (20.9%) and husbands (10%). There was no significant difference between the two groups in the use of information resources about pregnancy (p > 0.05).

## Discussion

The main objective of this study was to evaluate the effect of counseling on pregnant women’s anxiety in the third trimester of pregnancy. The results showed a significant reduction in state and trait anxiety levels of pregnant women in the experimental group after the intervention (p < 0.001); however, no significant difference was observed in state and trait anxiety levels of pregnant women in the control group after the intervention. These findings confirmed the effectiveness of counseling in reducing state and trait anxiety in pregnant women. Consulting with pregnant women and educating them during pregnancy can be effective in reducing pregnancy complications. Increasing awareness and preparedness training give pregnant women a chance to experience this phase of life with pleasure. Pregnancy is therefore an opportunity to educate women to have a healthier lifestyle (18). Alusen and colleagues (15) showed that childbirth education leads to reduced levels of anxiety in pregnant women, improves their mental health and enhances maternal-fetal attachment. Similarly, Parsa and colleagues (24) showed that pregnant women with lower levels of anxiety and higher levels of social support can experience a more successful breastfeeding. 

**Table 2:** Comparison of trait anxiety scores in two groups before and after the intervention

**Group**	**Before** **Mean ± SD**	**After** **Mean ± SD**	**Statistics (Paired t-test)**	**p**
Intervention	35.56 ± 3.74	30.75 ± 2.54	t = 11.983	< 0.001
Control	36.11 ± .93	38.33 ± 5.91	t = 1.901	0.06
Statistics (Independent t-test)	t = 0.75	t = 8.74		
P	0.457	< 0.001		

**Table3:** Comparison of state anxiety scores in two groups before and after the intervention

**Group**	**Before** **Mean ± SD**	**After** **Mean ± SD**	**Statistics (Paired t-test)**	**p**
Intervention	35.09 ± 4.03	29.96 ± 3.23	t = 15.196	< 0.001
Control	34.69 ± 4.33	38.44 ± 6.05	t = 1.802	0.08
Statistics (Independent t-test)	t = 0.502	t = 9.171		
P	0.617	< 0.001		

These findings were in line with results of the present study; however, the effectiveness of counseling in reducing anxiety varied across the mentioned studies that might be due to different counseling methods or different duration or timing of counseling sessions. Maternal-fetal attachment behaviors education leads to optimal relationships and compatibility between mothers and their fetuses. This type of education plays an important role in children’s future development. It has been indicated that maternal attachment is an essential factor in motherhood and that it positively affects the health of pregnant women and their fetuses (16, 17, 24).

In order to reduce women’s anxiety during pregnancy, they must be diverted from daily problems and conflicts. Thinking about the fetus and performing maternal-fetal attachment techniques (e.g. talking to the fetus and touching the abdomen) can draw mothers’ attention to more interesting issues. Daily repetition of such behaviors suppresses unwanted thoughts and reduces anxiety levels (25). Due to positive effects of enhanced maternal-fetal attachment on pregnant women’s mental health, they become motivated to better exercise pregnancy and childbirth health behaviors such as, proper nutrition, exercise, willingness to know the fetus and participation in pregnancy education programs, frequent breastfeeding, etc. Such behaviors lead to satisfactory pregnancy outcomes and improved health status of mothers and newborns (26). On the other hand, higher levels of anxiety and depression have been reported in mothers with lower levels of maternal attachment. Such problems can lead to adverse pregnancy outcomes for both mothers and newborns (27). Grizenko and colleagues (28) showed that children born to mothers with high levels of anxiety during pregnancy are prone to behavioral and psychological problems such as, ADHD and lower levels of IQ (29). Similarly, children suffering from excessive levels of stress feel less efficient in performing their tasks (30).

Among the strength of this study, the use of group counseling method can be mentioned. Pregnant women could make use of others’ experiences and better control their anxiety levels through group counseling sessions. Thus, it can be concluded that group counselingis an easy, inexpensive and non-invasive method to reduce pregnancy-related anxiety, especially in first-time mothers. Moreover, midwives can truly enhance behavioral health of pregnant women during their pregnancy. Performing relaxation and maternal-fetal attachmnet techniques not only reduces pregnancy-related concerns, but also enhances future health of mothers and newborns.

Attachment behaviors education can be used as an effective non-medicinal method in reducing anxiety. Reducing anxiety is an essential component of pregnancy care, especially for first-time mothers who are exposed to anxiety-provoking thoughts due to lack of knowledge and experience or exposure to public opinions. Anxious pregnant women must be identified by midwifes and nurses who are responsible for enhancing levels of maternal-fetal attachment through proper techniques such as listening to the fetus’ heartbeat or focusing on fetal movements. 

Among the limitations of the present study, examining only patients referring to one hospital can be mentioned, although, they were from every part of the city of Hamadan. Lack of access to husbands and not providing them with training programs may not helping them reduce anxiety of their pregnant wives were other limitations of the present study. Accordingly, childbirth preparation classes can be held for couples after making the necessary changes and modifying childbirth education programs.

## Conclusion

The results of this study showed that prenatal counseling can be used as an effective method to reduce pregnant women’s anxiety. Besides, properly educating health workers, especially midwives, about effective methods of consultation can help parents enhance parental-fetal attachment levels. According to the results of this study, midwives and other health workers having contact with pregnant women are recommended to support them during the prenatal period and thereby enhance mothers’ and newborns’ health status.
